# Predictors of the need for atrioventricular nodal ablation following redo ablation for atrial fibrillation

**DOI:** 10.1002/joa3.13023

**Published:** 2024-03-19

**Authors:** Peter Calvert, Wern Yew Ding, Michael Griffin, Arnaud Bisson, Ioanna Koniari, Noel Fitzpatrick, Richard Snowdon, Simon Modi, Vishal Luther, Saagar Mahida, Johan Waktare, Zoltan Borbas, Reza Ashrafi, Derick Todd, Archana Rao, Dhiraj Gupta

**Affiliations:** ^1^ Liverpool Centre for Cardiovascular Science at University of Liverpool, Liverpool John Moores University and Liverpool Heart & Chest Hospital Liverpool UK; ^2^ Liverpool Heart & Chest Hospital NHS Foundation Trust Liverpool UK; ^3^ Centre Hospitalier Régional Universitaire et Faculté de Médecine de Tours Tours France

**Keywords:** ablation, atrial fibrillation, atrioventricular nodal ablation, pace and ablate, pacemaker

## Abstract

**Background:**

Patients who have recurrent atrial fibrillation (AF) following redo catheter ablation may eventually be managed with a pace‐and‐ablate approach, involving pacemaker implant followed by *atrioventricular nodal ablation* (AVNA). We sought to determine which factors would predict subsequent AVNA in patients undergoing redo AF ablation.

**Methods:**

We analyzed patients undergoing redo AF ablations between 2013 and 2019 at our institution. Follow‐up was censored on December 31, 2021. Patients with no available follow‐up data were excluded. Time‐to‐event analysis with Cox proportional hazard regression was used to compare those who underwent AVNA to those who did not.

**Results:**

A total of 467 patients were included, of whom 39 (8.4%) underwent AVNA. After multivariable adjustment, female sex (aHR 4.68 [95% CI 2.30–9.50]; *p* < 0.001), ischemic heart disease (aHR 2.99 [95% CI 1.25–7.16]; *p* = 0.014), presence of a preexisting pacemaker (aHR 3.25 [95% CI 1.10–9.60]; *p* = 0.033), and persistent AF (aHR 2.22 [95% CI 1.07–4.59]; *p* = 0.032) were associated with increased risk of subsequent AVNA requirement.

**Conclusion:**

Female sex, ischemic heart disease, and persistent AF may be useful clinical predictors of the requirement for subsequent AVNA and may be considered as part of shared clinical decision making.

## Graphical abstract

Atrioventricular (AV) nodal ablation may be required for the treatment of atrial fibrillation (AF) when rhythm control measures fail. Our study found that female sex, ischemic heart disease, preexisting pacemaker, and persistent AF were independent predictors of the need for subsequent AV node ablation.

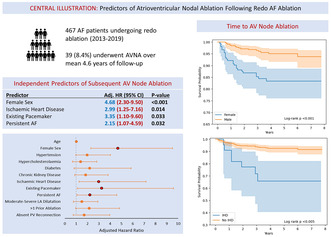

## 1 INTRODUCTION

In patients managed by a rhythm control strategy, catheter ablation for atrial fibrillation (AF) is a well‐established approach for maintaining sinus rhythm. Unfortunately, arrhythmia recurrence is not infrequently seen, particularly in more persistent forms of AF. In appropriately selected patients, a redo ablation procedure may be offered. A redo procedure carries the advantage of providing further arrhythmia suppression^1^; however, it may not always be successful.

Some patients progress to a stage of AF whereby attempts at rhythm control may no longer be appropriate because of the low chance of success. In such cases, especially where rate‐controlling drug therapy fails, the option of pacemaker implant followed by atrioventricular nodal ablation (AVNA) may be offered (“pace‐and‐ablate”). This approach guarantees rate control and regularity of ventricular rhythm but renders the patient dependent upon a pacemaker.

Determining the most appropriate strategy can, at times, be challenging, and some patients who undergo repeated AF ablations may eventually require AVNA. In this study, we sought to determine which factors may predict the subsequent choice to perform AVNA in a cohort of patients undergoing redo AF ablation.

## 2 METHODS

### 2.1 Study design and patient selection

This study was a single center, retrospective observational analysis of patients who underwent redo catheter ablation procedure(s) for AF between 2013 and 2019. Patients were identified from our institutional ablation dataset. No other inclusion criteria were applied; however, patients for whom no follow‐up was available were excluded. For patients with more than 2 ablations within the timeframe, we took the latest ablation to be the event of interest.

Identified records were manually reviewed to ensure accuracy of data, extract demographic and clinical data, and to determine follow‐up outcomes. Follow‐up was censored on December 31, 2021. The study was approved by our local Research & Innovation Committee.

### 2.2 Outcome measures

The outcome of interest in this analysis was the need for a pace‐and‐ablate approach after redo ablation. This was identified from electronic patient records as the date on which the patient underwent AVNA. All AVNA procedures were undertaken on clinical grounds, broadly either for resistant rate control, heart failure, or symptomatic arrhythmia paroxysms where further ablation and/or antiarrhythmic medications were not considered optimal.

We also performed a sub‐analysis including only those patients who suffered arrhythmia recurrence, comparing those undergoing subsequent AVNA with those who did not.

### 2.3 Statistical analysis

Continuous variables were expressed as mean ± standard deviation, or median [25th quartile–75th quartile] depending upon the distribution and compared using *t*‐tests or nonparametric equivalents. Categorical variables were expressed as counts and percentages and compared using Fisher's exact test. Time‐to‐event outcomes were analyzed using Cox proportional hazard regression and Kaplan–Meier plots. *p*‐Values <0.05 were considered statistically significant. Missing data were handled by multivariable imputation by chained equations. Statistical analysis was performed in Python and R.

## 3 RESULTS

### 3.1 Baseline characteristics

A total of 488 patients were identified, of whom 21 (4.3%) were excluded because of loss‐to‐follow‐up. Thus, a total of 467 patients met the inclusion criteria, of whom 39 (8.4%) underwent AVNA during mean follow‐up of 4.6 ± 1.7 years. Demographic and clinical differences between AVNA and non‐AVNA patients are shown in Table 1.

**TABLE 1:** Demographic and clinical differences between AVNA and non‐AVNA patients.

	AVNA (*n* = 39)	No AVNA (*n* = 428)	*p*‐Value
Age (median [IQR])	68 [61–71]	62 [55–69]	**0.002**
Female (%)	61.5	29.9	**<0.001**
BMI (mean ± SD)	30.2 ± 4.6	29.2 ± 5.1	0.214
Existing pacemaker	10.3	2.8	**0.036**
Persistent AF (%)	61.5	41.6	**0.018**
Months since prior ablation (median [IQR])	9 [4.5–12]	10 [5–21]	0.098
Hypertension (%)	61.5	37.4	**0.005**
Hypercholesterolemia (%)	48.7	31.3	**0.041**
Diabetes (%)	12.8	4.9	0.056
Chronic kidney disease (%)	25.6	13.3	0.062
Ischemic heart disease (%)	17.9	3.5	**0.001**
Heart failure (%)	10.3	4.9	0.146
Cerebrovascular disease (%)	12.8	7.9	0.357
CHA_2_DS_2_‐VASc score (median [IQR])	3 [2–3]	1 [0–2]	**<0.001**
Current antiarrhythmic drug use (%)	43.6	41.1	0.897
Significant^a^ LA dilatation (%)	43.6	26.2	**0.032**
Absent PV reconnection at redo (%)	23.1	9.1	**0.012**
>1 Prior AF ablation (%)	28.2	12.1	**0.010**
Prior AF ablations (median [IQR])	1 [1–2]	1 [1–1]	**0.004**

Abbreviations: AF, atrial fibrillation; AVNA, atrioventricular nodal ablation; BMI, body mass index; LA, left atrial; PV, pulmonary vein.

^a^ Significant defined as moderate or severe based on echocardiographic parameters.

Bold text highlights significant p‐values (i.e. <0.05).

Indications for AVNA broadly fell into three categories. Twenty‐two (56%) patients underwent AVNA for resistant rate control. Eleven (28%) had symptomatic heart failure as the indication. Eighteen (46%) had paroxysms of atrial arrhythmia as in the indication—in these cases, shared decision making was undertaken to discuss options such as antiarrhythmic medication or further redo ablation, and the decision was made for AVNA. Note that these numbers total >100% because some patients had more than one indication (e.g., heart failure and poor rate control).

AVNA patients tended to be older, were more often female, had more frequent persistent AF, and were more likely to have preexisting pacemakers. Co‐morbidities such as hypertension, hyperlipidemia and ischemic heart disease were also more common in the AVNA group, reflected in a higher median CHA_2_DS_2_‐VASc score (3 vs. 1; *p* < 0.001). Similarly, moderate or severe left atrial dilatation, absence of pulmonary vein reconnection at redo procedure, and a history of multiple prior ablations (2 or more) were more common in the AVNA cohort.

### 3.2 Predictors of AVNA

On univariable hazard regression, numerous factors including age, female sex, comorbidities, presence of a preexisting pacemaker, persistent AF, and multiple prior ablations were associated with increased risk of subsequent AVNA (Table 2).

**TABLE 2:** Cox proportional hazard regression for predictors of AVNA requirement.

Parameter	Univariable hazard ratio (95% CI)	*p*‐Value	Multivariable adjusted HR (95% CI)	*p*‐Value
Age (per year)	1.06 (1.02–1.10)	**0.002**	1.02 (0.98–1.06)	0.416
Female sex	3.58 (1.88–6.83)	**<0.001**	4.68 (2.30–9.50)	**<0.001**
BMI (per unit)	1.04 (0.98–1.10)	0.230		
Hypertension	2.54 (1.33–4.83)	**0.005**	1.97 (0.97–4.02)	0.062
Hypercholesterolemia	2.02 (1.08–3.79)	**0.028**	1.38 (0.68–2.81)	0.373
Diabetes	2.75 (1.07–7.02)	**0.035**	2.19 (0.82–5.84)	0.116
Chronic kidney disease	2.29 (1.11–4.70)	**0.024**	1.83 (0.87–3.82)	0.109
Ischemic heart disease	4.81 (2.12–10.90)	**<0.001**	2.99 (1.25–7.16)	**0.014**
Heart failure	2.09 (0.74–5.89)	0.162		
CHADSVASC score (per unit)	1.73 (1.42–2.11)	**<0.001**	^b^	
Existing pacemaker	3.84 (1.36–10.80)	**0.011**	3.25 (1.10–9.60)	**0.033**
Persistent AF	2.21 (1.16–4.23)	**0.016**	2.22 (1.07–4.59)	**0.032**
Significant^a^ LA dilatation	2.14 (1.13–4.03)	**0.019**	1.49 (0.76–2.91)	0.245
Months since prior ablation	0.98 (0.95–1.01)	0.104		
>1 Prior AF ablation	2.75 (1.37–5.53)	**0.004**	2.15 (0.96–4.82)	0.063
Absent PV reconnection	2.80 (1.33–5.89)	**0.007**	1.71 (0.74–3.97)	0.212

Abbreviations: AF, atrial fibrillation; AVNA, atrioventricular nodal ablation; BMI, body mass index; HR, hazard ratio; LA, left atrial; PV, pulmonary vein.

^a^ Significant defined as moderate or severe based on echocardiographic parameters.

^b^ CHADSVASC score was excluded from the multivariable model as a result of multicollinearity with the individual parameters of the model (e.g., hypertension, diabetes).

Bold text highlights significant p‐values (i.e. <0.05).

After multivariable adjustment, female sex (aHR 4.68 [95% CI 2.30–9.50]; *p* < 0.001), ischemic heart disease (aHR 2.99 [95% CI 1.25–7.16]; *p* = 0.014), presence of a preexisting pacemaker (aHR 3.25 [95% CI 1.10–9.60]; *p* = 0.033), and persistent AF (aHR 2.22 [95% CI 1.07–4.59]; *p* = 0.032) remained strongly associated with increased risk of subsequent AVNA.

**FIGURE 1:**
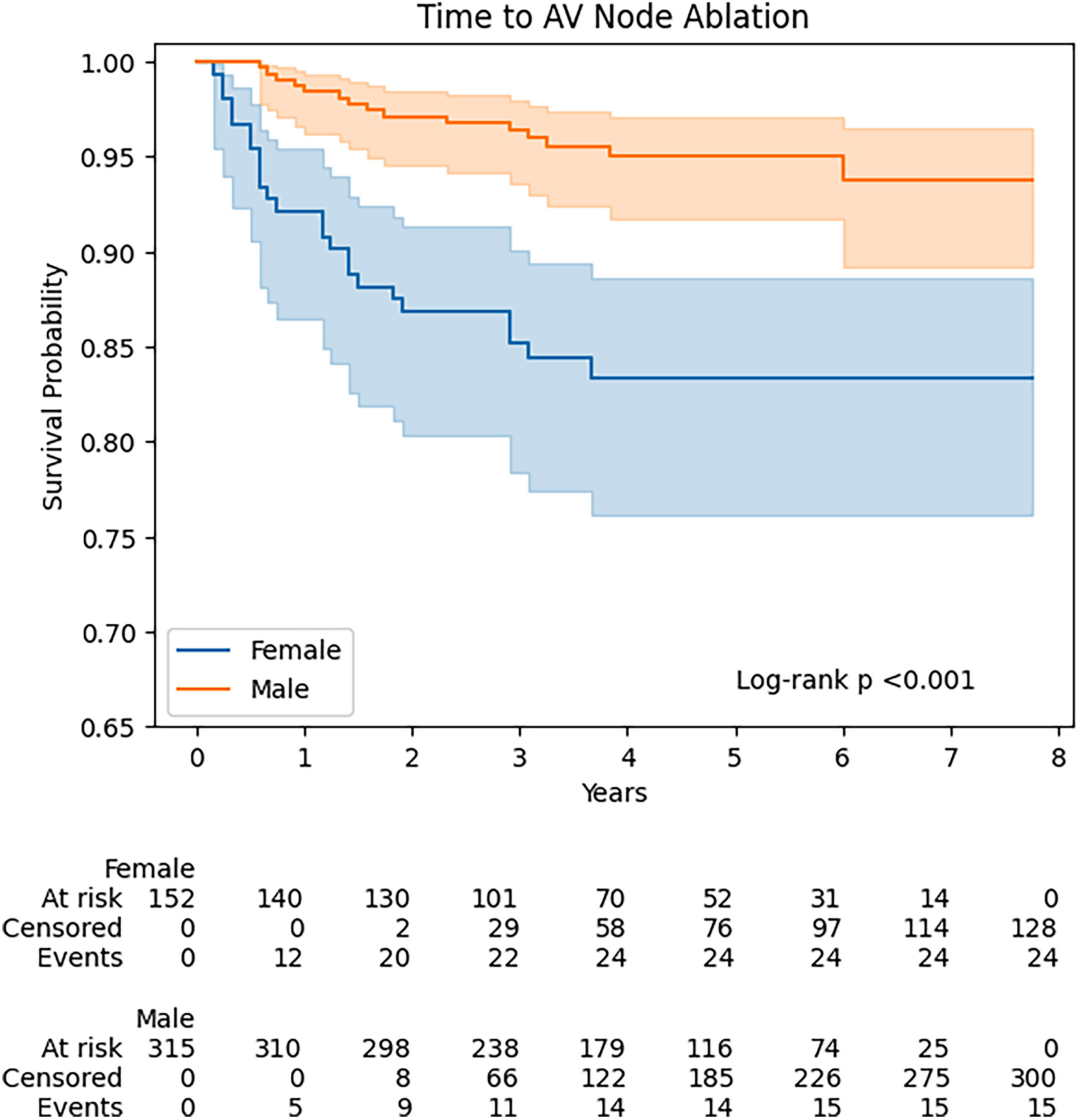
Kaplan–Meier plot for sex differences in time to atrioventricular (AV) node ablation.

**FIGURE 2:**
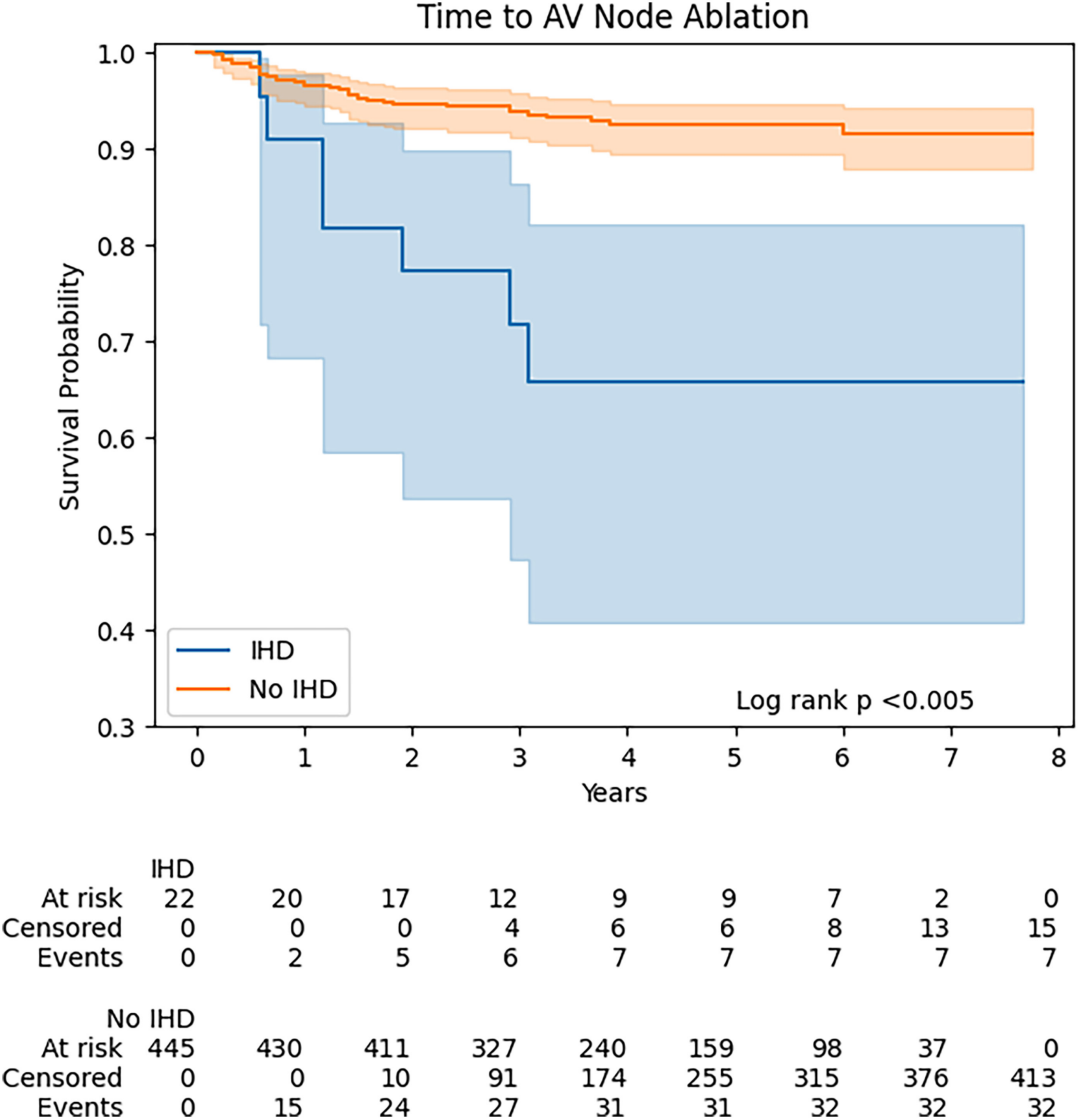
Kaplan–Meier plot for ischemic heart disease on time to atrioventricular (AV) node ablation. IHD, ischemic heart disease.

The proportional hazards assumption was met for the global model (Schoenfeld test *p* = 0.209). No interaction was found between female sex and ischemic heart disease (interaction *p* = 0.584), female sex and persistent AF (interaction *p* = 0.642), or ischemic heart disease and persistent AF (interaction *p* = 0.734). Kaplan–Meier plots for sex differences and ischemic heart disease are shown in Figures 1 and 2 respectively.

### 3.3 Sub‐analysis—Arrhythmia recurrence only

Of 467 patients meeting our inclusion criteria, 244 suffered arrhythmia recurrence. Of these, the same 39 (16%) patients all underwent AVNA, whilst 205 did not. Table 3 shows the demographic and clinical differences between these patient groups.

**TABLE 3:** Demographic and clinical differences between patients with arrhythmia recurrence with and without subsequent AVNA.

Arrhythmia recurrence only (*n* = 244)	AVNA (*n* = 39)	No AVNA (*n* = 205)	*p*‐Value
Age (median [IQR])	68 [61–71]	63 [55–69]	**0.008**
Female (%)	61.5	29.9	**<0.001**
BMI (mean ± SD)	30.2 ± 4.6	29.4 ± 5.0	0.349
Existing pacemaker	10.3	3.4	0.080
Persistent AF (%)	61.5	46.3	0.115
Months since prior ablation (median [IQR])	9 [4.5–12]	11 [6–23]	**0.016**
Hypertension (%)	61.5	41.5	**0.023**
Hypercholesterolemia (%)	48.7	34.6	0.105
Diabetes (%)	12.8	4.4	0.054
Chronic kidney disease (%)	25.6	12.2	**0.043**
Ischemic heart disease (%)	17.9	3.9	**0.004**
Heart failure (%)	10.3	4.9	0.249
Cerebrovascular disease (%)	12.8	5.9	0.161
CHA_2_DS_2_‐VASc score (median [IQR])	3 [2–3]	1 [1–2]	**<0.001**
Current antiarrhythmic drug use (%)	43.6	41.5	0.944
Significant^a^ LA dilatation (%)	43.6	33.2	0.271
Absent PV reconnection at redo (%)	23.1	11.2	0.066
>1 Prior AF ablation (%)	28.2	18.0	0.185
Prior AF ablations (median [IQR])	1 [1–2]	1 [1–1]	0.123

Abbreviations: AF, atrial fibrillation; AVNA, atrioventricular nodal ablation; BMI, body mass index; LA, left atrial; PV, pulmonary vein.

^a^ Significant defined as moderate or severe based on echocardiographic parameters.

Bold text highlights significant p‐values (i.e. <0.05).

Overall these two groups were similar to the main analysis, though several variables dropped under the level of statistical significance owing to the smaller number of overall patients. Months since prior ablation was statistically significant in this sub‐analysis (AVNA median 9 months vs. no AVNA median 11 months; *p* = 0.016).

## 4 DISCUSSION

In this study, our primary findings were that female sex, ischemic heart disease, persistent AF, and a preexisting pacemaker were strong independent predictors of subsequent AVNA. Sub‐analysis including only those with documented arrhythmia recurrence found similar trends to the primary analysis.

### 4.1 Female sex as a risk factor

Studies have shown that female patients suffer more AF‐related symptoms and consequently worse quality of life.^2^ This would suggest that restoring sinus rhythm would be more beneficial for female patients. Unfortunately, females may be less tolerant of antiarrhythmic drugs, have a higher recurrence rate following electrical cardioversion^3^ or ablation,^4^, ^5^ and are also at higher risk of complications from catheter ablation.^4^, ^5^, ^6^ There is even evidence that females may benefit more from a rate control approach.^7^

In addressing the question of why female sex may be a risk factor for subsequent AVNA, some studies show that females are referred later than males for AF management,^5^ resulting in a prolonged duration of AF and older age at the time of specialist review. This is important in an era where early rhythm control is gaining momentum, especially as there is evidence that women may need earlier implementation of rhythm control than their male counterparts.^8^

It is also recognized that females have smaller hearts, and more often develop diastolic impairment with aging.^9^ It is possible, therefore, that male patients with AF recurrence following ablation were more easily managed with rate‐controlling medication, whilst females required additional intervention to suppress symptoms.

### 4.2 Ischemic heart disease as a risk factor

There is evidence that coronary artery disease increases the risk of AF recurrence after electrical cardioversion,^3^ though at least one study found no difference in recurrence after catheter ablation.^10^ In the latter study, however, adverse clinical outcomes were more common in those with ischemic heart disease.

AF results in coronary blood flow abnormalities.^11^ It is therefore possible that those with ischemic heart disease are more likely to be symptomatic following AF recurrence, especially with poorly controlled rates. As ischemic heart disease and AF share common risk factors, which result in so‐called “atrial cardiomyopathy” and thus propagation of AF, these patients may be less likely to maintain sinus rhythm after redo procedures and thus may be more likely to receive AVNA on symptomatic grounds.

### 4.3 Other predictors

Presence of a preexisting pacemaker may guide clinical judgment in favor of AVNA when the clinical decision between further redo attempts and pace‐and‐ablate is less clear. A longstanding device, particularly when pacing parameters have been stable, provides reassurance that malfunction following AVNA is less likely to occur. Additionally, the presence of an existing pacemaker may reflect more advanced atrial disease, depending upon the implant indication.

The associations between AVNA, multiple prior ablations and absence of pulmonary vein reconnection are unsurprising. It is well established that pulmonary vein (PV) isolation is the predominant mechanism of benefit in AF ablation. A single redo procedure, allowing touch‐up ablation of PV reconnections, thereby provides additive benefit. Further ablations, however, are more likely to meet with “silent” PVs, in which case non‐PV triggers, suggestive of more advanced left atrial cardiomyopathy, may be causing recurrent arrhythmia. In this situation, there is significantly less evidence of benefit for further left atrial ablation.^12^ This is reflected in the univariable association between absent PV reconnection and subsequent AVNA (HR 2.80 [95% CI 1.33–5.89]; *p* = 0.007).

Similarly, the strong association of AVNA with persistent AF is not surprising. It is well established that persistent AF is more challenging to ablate, often associates with non‐PV triggers, and results in lower long‐term success rates than those for paroxysmal AF.

### 4.4 Risks of pace‐and‐ablate

One concern around pace‐and‐ablate is the risk of long‐term pacing resulting in pacing‐induced cardiomyopathy. This is well recognized with chronic right ventricular pacing. The fear of worsening heart failure may therefore push clinicians to pursue further attempts at left atrial ablation.

This risk has lessened in recent years with the advent of more advanced pacing systems, such as cardiac resynchronization therapy and conduction system pacing (CSP)—for example, His‐bundle pacing and left bundle area pacing.^13^ These advances, taken alongside the predictors described in our study, may help to avoid unnecessary attempts at redo AF ablation in settings where long‐term success is unlikely.

## 5 LIMITATIONS

Our study is subject to several limitations. Firstly, our data is observational in nature, and therefore unmeasured confounding is likely to be present. Secondly, our dataset was from a single institution based in the UK, which may not be generalizable to other institutions or countries. Thirdly, the number of AVNAs was relatively small which may result in underpowering for some associations.

Our study describes an outcome which was determined by physician judgment, and our timeframe pre‐dated the era of CSP. Hence, our findings may not accurately describe the evolving landscape of the pace‐and‐ablate approach. Clinician judgment may change over time as new evidence emerges and technology improves.

Additionally, our study describes associations with the need for AVNA, which are hypothesis generating and should not be interpreted as definitive causal links. Future prospective research would be beneficial to explore these associations further.

## 6 CONCLUSION

Female sex and ischemic heart disease may independently predict the need for subsequent AVNA in patients undergoing redo ablation for AF. Other relevant factors include advancing age, comorbidity burden, persistent AF, likelihood of non‐PV triggered AF, and presence of a well‐functioning existing pacemaker.

These factors may be considered as part of a shared decision‐making process, as in some cases it may be more efficacious and safer to proceed directly to pace‐and‐ablate without attempting redo catheter ablation.

## FUNDING INFORMATION

No funding was received for this work.

## CONFLICT OF INTEREST STATEMENT

DG reports: institutional research grants from Boston Scientific and Medtronic, and speaker fees from Boston Scientific. AB reports consultant or speaker fees from Medtronic. The other authors report no conflicts of interest.

## ETHICS STATEMENT

This study was approved by our local Research & Innovation Committee.

## CONSENT STATEMENT

N/A.

## CLINICAL TRIAL REGISTRATION

N/A.
